# Supplementary material for: Cross comparison of imaging strategies of mitochondria in C. elegans during aging
Source: bioRxiv. 2024 Dec 25:2024.12.24.630282. Preprint. [Version 1] doi: 10.1101/2024.12.24.630282 (PMC11703187; doi:10.1101/2024.12.24.630282)

bioRxiv preprint doi: https://doi.org/10.1101/2024.12.24.630282; this version posted December 25, 2024. The copyright holder for this preprint (which was not certified by peer review) is the author/funder, who has granted bioRxiv a license to display the preprint in perpetuity. It is made available under aCC-BY 4.0 International license.

Fig. S1. Comparative imaging of muscle mitochondria across strains. (A) Comparison of day 1 imaging of mosSCI MLS::GFP (RHS191) strain to multi-copy *myo-3::GFP(mit)* (SJ4103) and multi-copy *myo-3p::TOM20::mRFP* (PS6192) showing variability in SJ4103 and PS6192 but not in RHS191. Images are intentionally shown zoomed out to display multiple muscle cells per image. (**B**) Imaging of muscle MLS::GFP x TOMM-20::mCherry from the Mair lab at day 5 of adulthood. Arrowheads indicate outer membrane mCherry signal in the absence of matrix MLS::GFP. (**C**) Imaging of muscle MLS::mRuby during aging. All animals were grown on ev from the L1 stage and imaged at the indicated days.

Fig. S2. Comparison of manual manipulation of worms versus no manipulation of worms. Animals expressing tissue-specific MLS::GFP were grown on ev from the L1 stage. At day 1 of adulthood, animals were either moved onto plates supplemented with 100 µL of 10 mg/mL FUDR directly onto the food source, or on standard RNAi plates. Animals on standard RNAi plates and FUDR plates were moved daily (manual and FUDR+manual) or left undisturbed (FUDR). Imaging was performed at day 1, 5, 9, and 13 of adulthood in the **(A)** muscle, **(B)** intestine, and **(C)** hypodermis.

Fig. S3. MLS::GFP expression only has mild impacts on lifespan. Lifespans were performed on wild-type control (N2), and strains expressing MLS::GFP in the muscle (*myo-3p*), intestine (*vha-6p*), and hypodermis (*col-19p*), grown on ev from the L1 stage. All replicates with worms n > 90. Graphs were plotted and statistically analyzed by log-rank test (Mantel-Cox), using GraphPad Prism 10.0. ns = not significant, * = p < 0.03; ** = p <0.002; *** = p<0.0002; **** = p< 0.0001. All lifespan statistics are available in **Table S2**.

Table S1. Table of lifespan statistics.

bioRxiv preprint doi: https://doi.org/10.1101/2024.12.24.630282; this version posted December 25, 2024. The copyright holder for this preprint (which was not certified by peer review) is the author/funder, who has granted bioRxiv a license to display the preprint in perpetuity. It is made available under aCC-BY 4.0 International license.

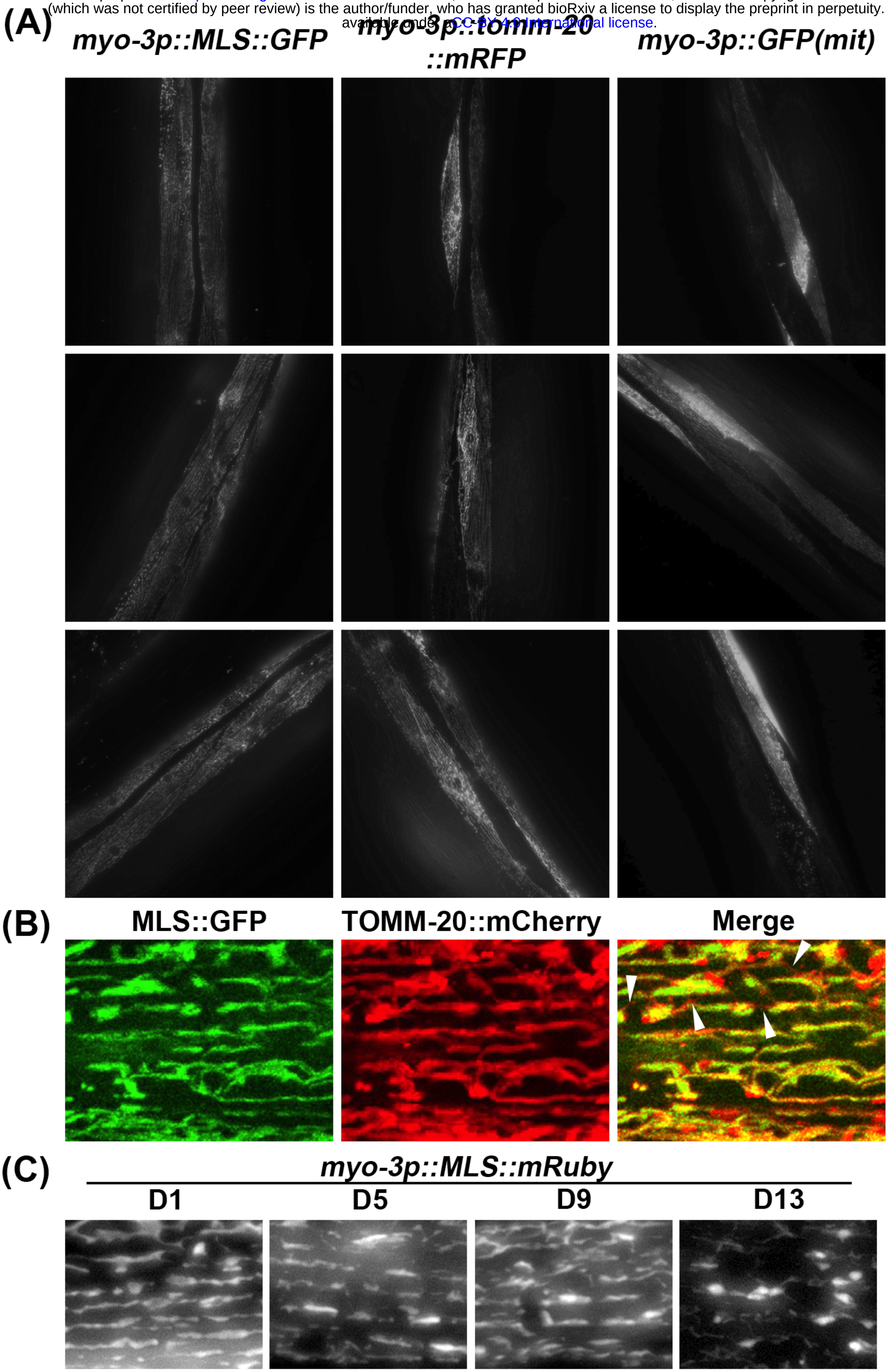

bioRxiv preprint doi: https://doi.org/10.1101/2024.12.24.630282; this version posted December 25, 2024. The copyright holder for this preprint (which was not certified by peer review) is the author/funder, who has granted bioRxiv a license to display the preprint in perpetuity. It is made available under aCC-BY 4.0 International license.

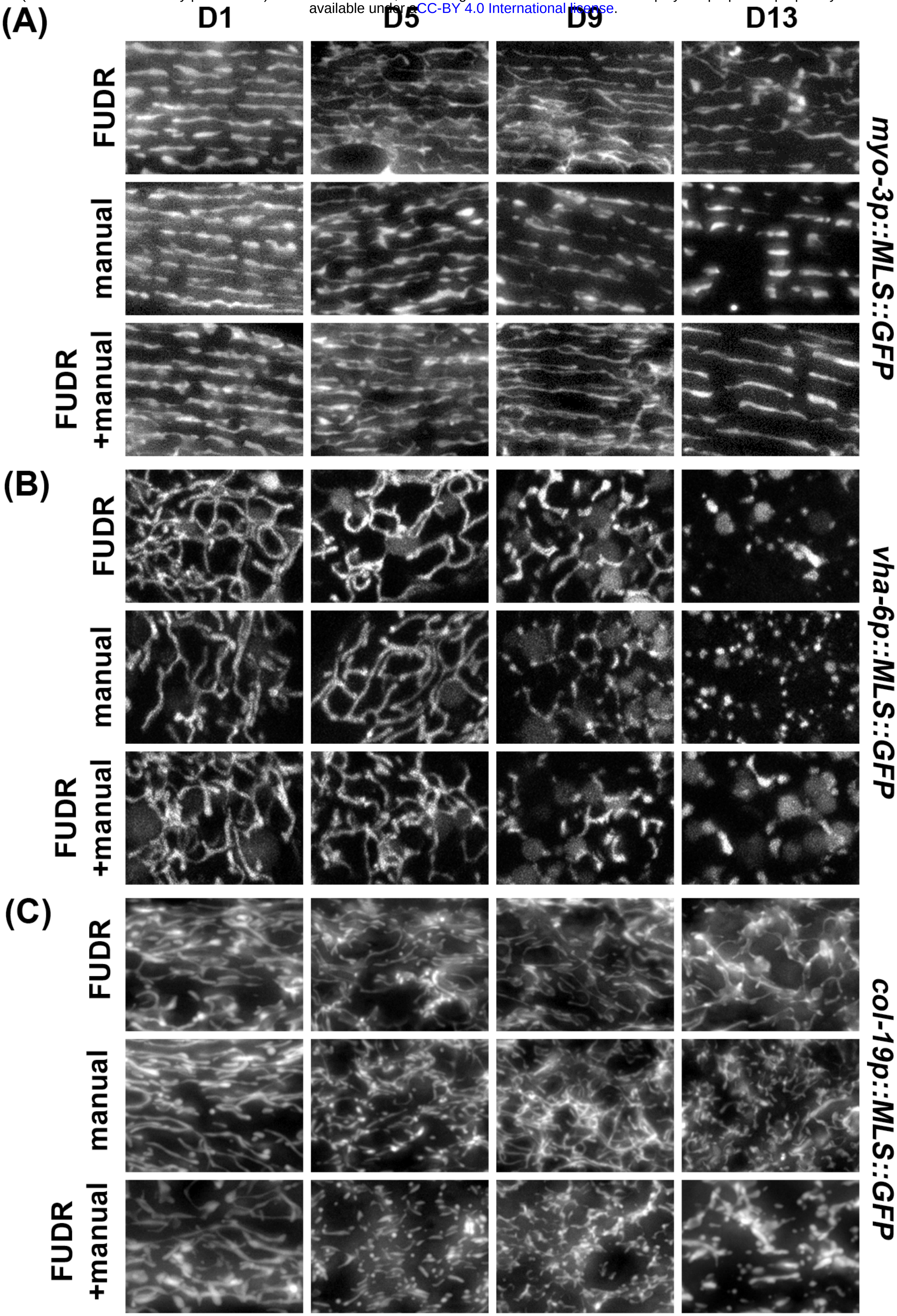

bioRxiv preprint doi: https://doi.org/10.1101/2024.12.24.630282; this version posted December 25, 2024. The copyright holder for this preprint (which was not certified by peer review) is the author/funder, who has granted bioRxiv a license to display the preprint in perpetuity. It is made available under aCC-BY 4.0 International license.

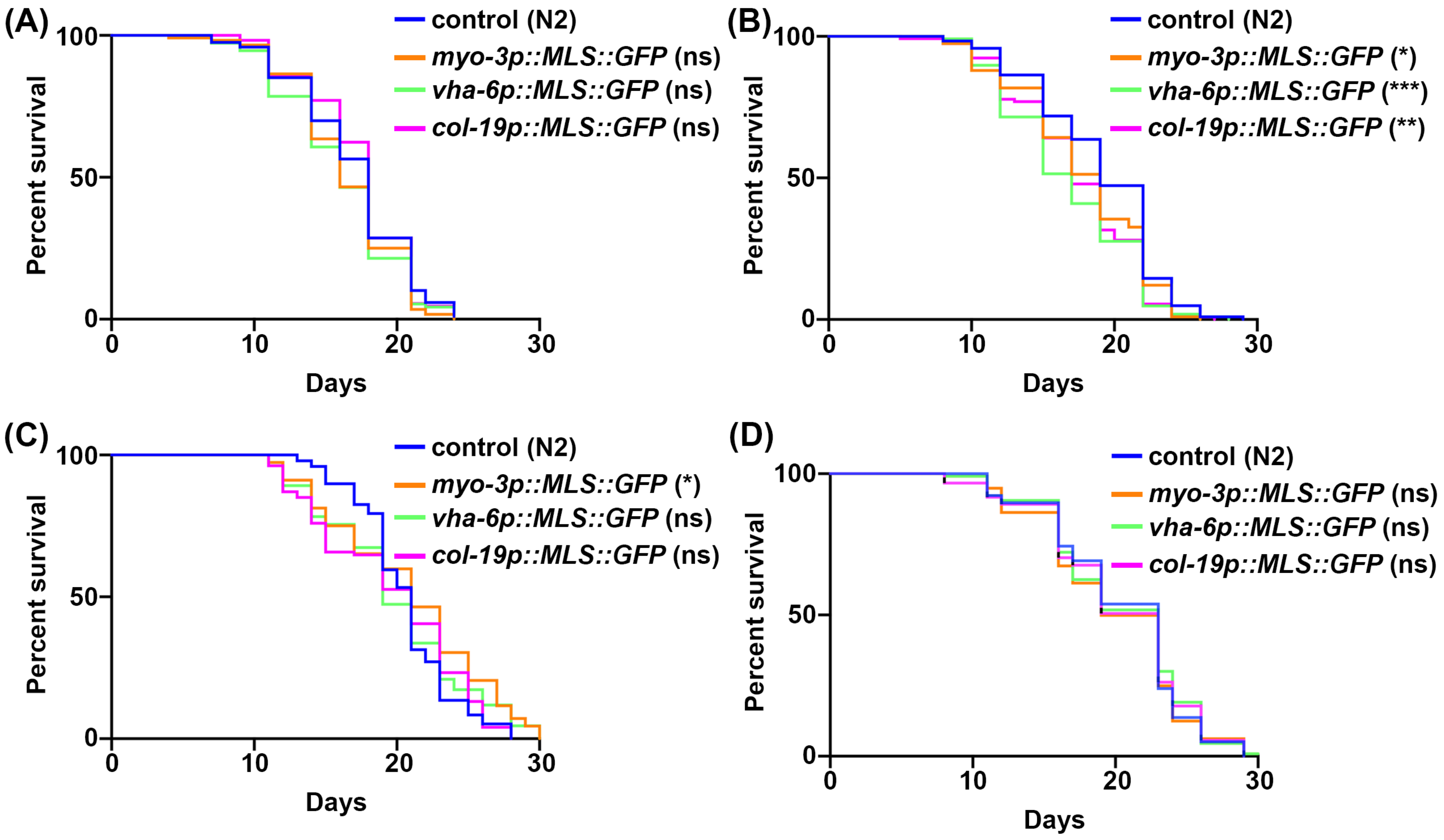

Supplement: 1 [file NIHPP2024.12.24.630282V1-supplement-1.pdf]
